# Carbohydrate Supplementation Influences Serum Cytokines after Exercise under Hypoxic Conditions

**DOI:** 10.3390/nu8110706

**Published:** 2016-11-05

**Authors:** Aline Venticinque Caris, Edgar Tavares Da Silva, Samile Amorim Dos Santos, Fabio Santos Lira, Lila Missae Oyama, Sergio Tufik, Ronaldo Vagner Thomatieli Dos Santos

**Affiliations:** 1 Department of Psychobiology, Universidade Federal de São Paulo, São Paulo 04021-001, Brazil; alinecaris@hotmail.com; 2 Department of Bioscience, Universidade Federal de São Paulo, Santos 11060-001, Brazil; edgartavares@uol.com.br (E.T.D.S.); samile.unifesp@gmail.com (S.A.D.S.); sergiotufik@zipmail.com.br (S.T.); 3 Department of Physical Education, Universidade Estadual Paulista, Presidente Prudente 19060-900, Brazil; fslira@gmail.com; 4 Department of Physiology, Universidade Federal de São Paulo, São Paulo 04021-001, Brazil; lmoyama@gmail.com

**Keywords:** supplementation, carbohydrate, hypoxia, exercise, immune system, IL-2/IL-4

## Abstract

Introduction: Exercise performed at the hypoxia equivalent of an altitude of 4200 m is associated with elevated inflammatory mediators and changes in the Th1/Th2 response. By contrast, supplementation with carbohydrates has an anti-inflammatory effect when exercise is performed under normoxic conditions. The objective of this study was to evaluate the effect of carbohydrate supplementation on cytokines and cellular damage markers after exercise under hypoxic conditions at a simulated altitude of 4200 m. Methods: Seven adult male volunteers who exercised for 60 min at an intensity of 50% VO_2Peak_ were randomly evaluated under three distinct conditions; normoxia, hypoxia and hypoxia + carbohydrate supplementation. Blood samples were collected at rest, at the end of exercise and after 60 min of recovery. To evaluate hypoxia + carbohydrate supplementation, volunteers received a solution of 6% carbohydrate (maltodextrin) or a placebo (strawberry-flavored Crystal Light^®^; Kraft Foods, Northfield, IL, USA) every 20 min during exercise and recovery. Statistical analyses comprised analysis of variance, with a one-way ANOVA followed by the Tukey post hoc test with a significance level of *p* < 0.05. Results: Under normoxic and hypoxic conditions, there was a significant increase in the concentration of IL-6 after exercise and after recovery compared to at rest (*p* < 0.05), while in the hypoxia + carbohydrate group, there was a significant increase in the concentration of IL-6 and TNF-α after exercise compared to at rest (*p* < 0.05). Furthermore, under this condition, TNF-α, IL-2 and the balance of IL-2/IL-4 were increased after recovery compared to at rest (*p* < 0.05). Conclusion: We conclude that carbohydrate supplementation modified the IL-6 and TNF-α serum concentrations and shifted the IL-2/IL-4 balance towards Th1 in response without glycemic, glutaminemia and cell damage effects.

## 1. Introduction

Hypoxia is the main characteristic of the body’s response to high altitudes and diseases, such as apnea and chronic obstructive pulmonary disease (COPD), and can be a challenge to the body due to the stress it produces on various physiological systems [1]. However, because few studies have been conducted under hypoxic conditions for the specific purpose of monitoring different immune parameters in humans, the effects remain unclear [2]. Several recent studies have suggested that hypoxia may modulate important aspects of the immune response, inflammation and metabolism [3,4]. For example, hypoxia at high altitude is associated with elevated levels of inflammatory mediators [5,6,7].

Exhaustive physical exercise induces acute inflammation in healthy individuals, an event that can promote tissue repair and remodelling after cell damage caused during exercise [8,9]. Another effect of physical exercise on the immune system is displayed in the cellular and humoral responses, as indicated by changes in the Th1/Th2 cytokines, including IL-2, IL-4, IL-6, TNF-α and INF-γ, which are dependent on the intensity and duration of exercise [10].

When exercise is performed under hypoxic conditions, it can generate a different and more intense response than that generated by exercise performed under normoxia [11]; however, the effects of exercise on immune function and cytokines under hypoxic conditions remain unclear [12]. Caris et al. [13] showed that physical exercise performed on a treadmill at 70% VO_2peak_ until maximum voluntary exhaustion at a simulated altitude of 4500 m shifts the Th1/Th2 balance towards the Th2 response after exercise.

Many studies have shown that carbohydrate supplementation during and after exercise in normoxia affects immune cell function, serum cytokine and myokine expression, promoting anti-inflammatory and immunostimulating effects [14,15,16,17]. These findings suggest that carbohydrate supplementation may be a useful strategy to attenuate the inflammatory and immunosuppressive effects of hypoxia. Based on the above, the objective of this study was to evaluate the effect of carbohydrate supplementation on serum cytokine concentration after exercise under hypoxic conditions.

## 2. Methods

### 2.1. Participants

The present study was comprised of 7 male volunteers who were healthy and physically active (performing aerobic exercise at least three times a week for at least one year) and with similar physiological and anthropometric characteristics as follows: age (years) 23 ± 2; body weight (kg) 73.47 ± 9.42; height (m) 171.19 ± 5.74; BMI (kg/m^2^) 22.69 ± 3.07; VO_2peak_ (mL/kg/min) 48.80 ± 4.12; velocity maximal (km/h) 16.93 ± 2.19; time to exhaustion (min) 14.47 ± 1.66. Volunteers with health problems or others issues that could influence the results of the study were excluded. These included changes in the electrocardiogram (ECG) at rest and effort after clinical evaluation conducted by a doctor that prevented the performance of physical exercise, smokers and/or drug abusers, those who used any medication that could interfere with the study results, those who used alcoholic beverages frequently (more than three times per week) and those who had been exposed to an altitude of over 1500 m during the previous six months.

The Ethics Committee for Research of the Federal University of São Paulo (UNIFESP) (CEP-0620/09) approved the study protocol in accordance with the guidelines of the 1964 Declaration of Helsinki. All participants gave written informed consent.

### 2.2. Experimental Design

The volunteers visited the laboratory five times, with an interval of 7 days between each session. During the first session, comprehensive information on the objectives and procedures of the study was provided to the volunteers. The volunteers then signed an informed consent form. After signing the form, the volunteers underwent an electrocardiogram at rest and after effort. In the second session, they underwent an ergospirometry test with progressive intensity until volitional exhaustion to determine peak oxygen consumption (VO_2peak_) under normoxic conditions. In the other sessions, the volunteers underwent the following procedures: (I) exercise at normoxia without supplementation (placebo); (II) exercise at hypoxia without supplementation (placebo); and (III) exercise at hypoxia with carbohydrate supplementation (maltodextrin). All procedures were double blind and randomized in regards to supplementation.

### 2.3. Determination of VO_2peak_

The peak oxygen consumption (VO_2peak_) for each volunteer was determined under normoxic conditions using an incremental exercise test on a treadmill (LifeFitness^®^—9700HR, Rosemont, IL, USA). The initial velocity was set at 6.0 km/h, and the speed was increased by 1.0 km/h per minute until voluntary exhaustion. Respiratory and metabolic variables were obtained for each breath by measuring the gaseous respiratory exchanges with a metabolic system (COSMED PFT4, Rome, Italy). Voluntary exhaustion was defined as the inability to sustain the speed of the treadmill for 15 seconds or until the volunteers asked to stop the test even after being encouraged to continue [18]. Throughout the test, we used a fixed inclination of 1%. 

### 2.4. Simulation of Hypoxia

The study was performed in a chamber (normobaric chamber; Colorado Altitude Training/12 CAT-Air Unit) for altitude simulations of up to 4200 m, which is equivalent to a barometric pressure of 464 mmHg and a fraction of inspired oxygen (FiO_2_) of 13.5% O_2_. This is considered an altitude at which the effects of hypoxia become more evident.

### 2.5. Physical Exercise and Recovery

The exercise was performed with an intensity equivalent to 50% VO_2peak_ for 60 min under normoxia or hypoxia. This overload of exercise was chosen to induce changes in cytokines and increase the importance of carbohydrate metabolism. All sessions of physical exercise were performed after fasting for three hours to avoid possible dietary influences, and the tests began at 2:00 p.m. A 60-min recovery period after the exercise was performed under normoxia.

### 2.6. Supplementation

For the hypoxia trials, the volunteers received 200 mL of carbohydrate solution (maltodextrin) at 6% (w/v), every 20 min, or a placebo (strawberry-flavored Crystal Light^®^; Kraft Foods, Northfield, IL, USA), starting at the 20th min of exercise and continuing until the end of the 1 h during recovery. The groups received the same volume of solution containing carbohydrate or placebo in a double-blinded manner. The energy content of the placebo was 0 kcal and of the carbohydrate solution was 228 kcal.

### 2.7. Hemoglobin Oxygen Saturation

At rest, after exercise and one hour after exercise (recovery), oxygen saturation was assessed noninvasively. This was measured using a finger oximeter, Fingertrip^®^ (Nonim, Plymouth, IL, USA).

### 2.8. Blood Collection

At rest, after exercise and one hour after exercise, 20 mL of venous blood were collected. Blood samples were collected from the antecubital vein using dry glass tubes Vacutainer^®^ (BD, Franklin Lakes, NJ, USA), After collection, the blood was centrifuged at 690× *g* for 15 min at 4 °C, and the serum was then extracted and aliquoted for subsequent analysis. Samples were kept at −80 °C and analyzed within 2 months.

### 2.9. Serum Determinations

The concentrations of IL-2, IL-4, IL-6 and TNF-α were measured using commercially available ELISA-kits from R & D Systems^®^ (Minneapolis, MN, USA) to determine the Th1/Th2 response and cytokine profiles. Due to the relationship between glycemia and cytokine production during exercise, glucose was measured using kits from Bioclin^®^ (Quibasa, São Paulo, Brazil). Creatine kinase (CK), Creatine Kinase Muscle B (CK-MB) and lactate dehydrogenase (LDH) were determined enzymatically using kits from Bioclin^®^ (Quibasa, São Paulo, Brazil) and used as cellular damage markers. In addition, because of its immunomodulatory effect, the concentration of glutamine was determined enzymatically using commercial kits from Sigma^®^ (Saint Louis, MO, USA). All determinations were performed in accordance with the manufacturers’ instructions. Samples were run in duplicate, and all intra-assay coefficients of variation were less than 10%.

### 2.10. Statistical Analysis

The results are expressed as the mean ± standard deviation. Data normality was verified by the Shapiro–Wilk test. The data were normalized by Z-score when necessary, and statistical analyses were performed using two-way ANOVA followed by Tukey post hoc test with a significance level of *p* < 0.05. All data were analyzed using GraphPad Prism (Version 5.0, GraphPAD Software, LaJolla, CA, USA).

## 3. Results

Table 1 shows O_2_ saturation in the arterial blood (SaO_2_%) for the three conditions and three time points studied. There was a difference in the interaction between time point and trial condition (*F* = 11.12 and *p* < 001). For SaO_2_%, there was a significant difference between the time point (*F* = 38.4 and *p* = 0.001) and trial condition (*F* = 13.54 and *p* = 0.001). There was a decrease after exercise in relation to rest (*p* < 0.05), and there was an increase in recovery compared to recovery under hypoxic conditions (*p* < 0.05). When the volunteers were supplemented with carbohydrates, the SaO_2_% decreased after exercise in relation to rest (*p* < 0.05). Under normoxic conditions, there was no difference. In addition, SaO_2_% decreased after exercise in relation to normoxia (*p* < 0.05). 

Table 2 shows total CK, CK-MB and LDH. In relation to total CK, there was a significant difference in the time point (*F* = 14.28 and *p* = 0.001) and trial condition (*F* = 3.4 and *p* = 0.04). Under hypoxic conditions, there was an increase in total CK after exercise compared to rest (*p* < 0.05). There was a significant difference in CK-MB in the time point (*F* = 8.71 and *p* < 0.001) and trial condition (*F* = 4.37 and *p* = 0.01). In relation to LDH, there was a difference in the interaction between time point and trial condition (*F* = 4.45 and *p* = 0.003) and time point (*F* = 7.91 and *p* = 0.01). However, the serum CK-MB and LDH do not differ between trial condition and time point (Table 2).

Table 3 shows cytokine concentrations. For IL-6, there was difference in the time point (*F* = 42.95 and *p* = 0.001) and trial condition (*F* = 12.72 and *p* = 0.001). In addition, there was an increase after exercise and after recovery in relation to rest under normoxic and hypoxic conditions (*p* < 0.05). Additionally, carbohydrate supplementation promotes an increase of IL-6 concentrations after exercise compared to those at rest (*p* < 0.05). For TNF-α concentration, there was difference in the time point (*F* = 10.31 and *p* < 0.001) and trial condition (*F* = 4.58 and *p* = 0.01). There was an increase after exercise and after recovery compared to at rest for the hypoxia + carbohydrate conditions (*p* < 0.05). In relation to IL-10 concentration, there was difference in the time point (*F* = 7.59 and *p* = 0.001) and trial condition (*F* = 3.7 and *p* = 0.03). In relation to the TNF-α /IL-10 ratio, there was a difference in the interaction between time point and trial condition (*F* = 13.18 and *p* = 0.001), in the time point (*F* = 60.36 and *p* = 0.001) and trial condition (*F* = 16.59 and *p* = 0.001). 

In relation to IL-2, there was a difference in the time point (*F* = 4.28 and *p* = 0.01) and trial condition (*F* = 4.18 and *p* = 0.02). The IL-2/IL-4 ratio was different in the interaction between time point and trial condition (*F* = 4.10 and *p* = 0.005), in the time point (*F* = 6.04 and *p* = 0.004) and trail condition (*F* = 8.27 and *p* = 0.007). In addition, Table 4 shows an increase in IL-2 and the IL-2/IL-4 ratio in recovery compared to those at rest and after exercise for the hypoxia + carbohydrate condition (*p* < 0.05). The IL-2/IL-4 ratio was lower in hypoxia + carbo in relation to normoxia and hypoxia (*p* < 0.05).

In relation to glutamine, there was a difference in the interaction between time point and trial condition (*F* = 5.75 and *p* < 0.001), in the time point (*F* = 9.46 and *p* < 0.001) and trial condition (*F* = 5.90 and *p* < 0.001). The glutamine concentration decreased after exercise and recovery in relation to that observed at rest (*p* < 0.05) for normoxia. The glucose concentration was different in the interaction between time point and trial condition (*F* = 4.54 and *p* < 0.01) and trial condition (*F* = 2.54 and *p* < 0.02). Both glutamine and glucose are presented in Table 5.

## 4. Discussion

High altitudes are characterized by hypoxic environments [4,19], which can promote increased inflammatory cytokine release by skeletal muscle [20,21] and modify the immune and inflammatory responses [3]. Hypoxia results in stress to the body. In addition, exercise may cause a higher degree of immunosuppression [11].

However, under normoxia, nutritional strategies can be effective in mitigating the harmful effects of strenuous exercise on the body [22]. Therefore, the objective of this study was to evaluate the effects of carbohydrate supplementation on serum cytokines after exercise at a simulated altitude of 4200 m. The main result of the present study was that supplementation reduced the inflammatory response after exercise and changed the IL-6 and TNF-α concentrations. Furthermore, it increased IL-2 and the IL-2/IL-4 ratio, suggesting the regulation of the Th1/Th2 balance.

A decrease in inspired O_2_ is the main characteristic of the body’s response to hypoxic environments [23]. In both of the two hypoxic conditions, we found a reduction in hemoglobin oxygen saturation (SaO_2_%) from baseline, confirming that volunteers in this study were subjected to a hypoxic environment and supporting the findings of Pomidori et al. [24]. A few studies have shown that carbohydrate ingestion can improve blood oxygenation during exposure to acute hypoxia [25,26]. We observed that SaO_2_% was reduced after exercise under hypoxic conditions compared to baseline and recovery after 60 min of rest. Moreover, in the hypoxia + carbohydrate condition, no difference was found after recovery compared to baseline. Interestingly, the hypoxic condition reduced SaO_2_% by 8.2% after exercise, followed by an increase of 8.9% after recovery. By contrast, for the hypoxia + carbohydrate condition, the decrease was 5.2% and the increase was 3.3%, suggesting that carbohydrate supplementation helped lower SaO_2_% oscillation during exercise and recovery.

In the present study, exercise under normoxic conditions promoted an increase in serum IL-6, confirming previous studies [20,27]. Skeletal muscle produces IL-6 to maintain glucose metabolism [28]. When exercise is performed under hypoxia, it can be more physiologically stressful than exercise under normoxia [11]. Interestingly, despite the increase in IL-6 concentration for the hypoxic condition after exercise and recovery, we did not observe any difference for normoxia. Moreover, carbohydrate supplementation restored the IL-6 concentration after recovery. Our results are consistent with a recent study by Caris et al. [13], in which changes in the IL-6 concentration were reversed after 2 h recovery at 4500 m following exercise and recovery combined with the consumption of 8% maltodextrin every 20 min.

Carbohydrate supplementation can attenuate the increase in cortisol during exercise [16,20] and spare muscle glycogen [29], contributing to faster restoration of serum IL-6 during recovery. Another possibility may be that a lower oscillation of SaO_2_% under supplemented conditions has only a minor effect on O_2_ transport, requiring a weaker hematopoietic response by IL-6 [30]. This is described by Klausen et al. [31], who suggested that hypoxic environments modulate the production of cytokines, particularly IL-6.

TNF-α is one of the major mediators of the inflammatory response, capable of modulating inflammation by activation of NFkB and responsible for many systemic effects [32,33]. There was no change in serum TNF-α under hypoxic conditions, which can be explained by the increase of IL-6 that inhibits TNF-α production [34]. These results are consistent with the study by Caris et al. [13], who found similar changes after exercise at 70% VO_2peak_ in hypoxia simulation at 4500 m. In addition, Blegen et al. [35] tested two exercise intensities (40% VO_2_ max and 60% VO_2_ max) for 60 min in both normoxic (PiO_2_ = 20.94%) and hypoxic (PiO_2_ = 14.65%, equivalent ~4000 m) conditions and found no difference in TNF-α expression. Interestingly, for the carbohydrate supplemented condition, the increase in IL-6 during exercise did not reverse the increase of TNF-α at the end of exercise and recovery.

The reasons for the increase in TNF-α in the hypoxic condition supplemented with carbohydrates are not known. We propose that carbohydrate supplementation increases the glycolytic pathway products, particularly pyruvate and lactate, during exercise [36]. Combined with the hypoxic environment [33,37], the increased activity of the glycolytic pathway may inhibit HIF-1 degradation (hypoxia-inducible fator-1) in the cytosol [38]. Thus, activated HIF-1 migrates to the cell nucleus and acts as an essential transcription factor that connects the inflammatory pathways and stimulates serum TNF-α production [39].

IL-10 is an anti-inflammatory cytokine that is essential for the control and resolution of the inflammatory process initiated and maintained by other mediators [40]; therefore, the TNF-α/IL-10 ratio is indicative of the pro/anti-inflammatory balance. In our study, there was no change in the TNF-α/IL-10 ratio and IL-10 concentration for any of the evaluated conditions. It is possible that the changes in IL-6 and TNF-α were not sufficient to promote an imbalance in the pro-/anti-inflammatory balance. This may be different based on the exercise intensity or exposure time [41,42] and the magnitude of hypoxia [43]. For example, if hypoxia levels were higher and/or maintained longer, supplementation may be beneficial to the individual. In addition, stored glycogen, nutritional status and temperature are others possible mechanisms that may explain changes in cytokine production.

Moderate exercise increases the IL-2 levels that may modulate the immune response mediated by cells [10], and our results demonstrate that IL-2 was increased by 57.1% under normoxia after a 60-min recovery period, whereas under hypoxia, the IL-2 concentration was 57.4% lower at the same time. This contrasts with the hypoxia + carbohydrate condition, in which there was a significant increase in the IL-2 concentration after recovery. This change in the IL-2 level may have occurred in synergy with the increased TNF-α, which correlates with the Th1 immune response [15,44].

The modified IL-2 after recovery under the supplemented condition induced an increase in the IL-2/IL-4 ratio, suggesting a shift in the profile towards the Th1 response. A similar response was found in the study by Caris et al. [13] and suggests that the supplementation used in our study has a similar effect in hypoxia and normoxia as confirmed by previous studies [45]. This result contrasts with the decrease of 41% in the IL-2/IL-4 ratio found after recovery under the hypoxic condition without supplementation.

Various studies have suggested the importance of glucose and glutamine in the regulation of serum cytokines, immune function and inflammatory response at rest [46,47] and post exercise [44,48]. Other studies have shown the relationship between cell injury markers and inflammation after exercise [49,50]. In our study, we observed no change in CK, LDH, glucose and glutamine concentration, suggesting that under the conditions studied, other mechanisms mediate cytokine changes.

Because of the consequences of exposure to high altitude environments and because the number of people who ascend to these regions each year increases, our group conducted the first study to elucidate the effects of carbohydrate supplementation on cytokines after exercise under hypoxia. From this study, new interventions may be proposed and designed to minimize the effects of hypoxia for athletes, travelers, workers and people chronically exposed to hypoxia or suffering from diseases that worsen O_2_ saturation, such as sleep apnea and lung diseases.

## 5. Conclusions

We conclude that carbohydrate supplementation modifies the serum concentration of IL-6 and TNF-α and contributes to shifting the Th1/Th2 balance toward the Th1 response without cell damage, changes in blood glucose or glutaminemia.

## Figures and Tables

**Table 1:** O_2_ saturation percent (SaO_2_%) at rest, after exercise and after recovery in normoxia, hypoxia and hypoxia + carbo.

Condition	Rest	Exercise	Recovery
Normoxia	98 ± 1	96 ± 1	96 ± 2
Hypoxia	97 ± 1	90 ± 2 ^a,c^	97 ± 1 ^b^
Hypoxia + Carbo	96 ± 2	93 ± 2 ^a^	94 ± 2

Hemoglobin oxygen saturation (SaO_2_%) at rest, after exercise and after 60 min of recovery, in normoxia, hypoxia and hypoxia + carbohydrate for *n* = 7 volunteers. Values shown represent the mean ± SD. *p* < 0.05. ^a^ Different in relation to rest; ^b^ different in relation to exercise; and ^c^ different in relation to normoxia.

**Table 2:** Cellular damage at rest, after exercise and after recovery in normoxia, hypoxia and hypoxia + carbo.

	Condition	Rest	Exercise	Recovery
**Total CK**	Normoxia	107 ± 10	178 ± 50	147 ± 19
	Hypoxia	103 ± 35	210 ± 91 ^a^	157 ± 87
	Hypoxia + Carbo	81 ± 36	159 ± 83	110 ± 52
**CK-MB**	Normoxia	42 ± 4	71 ± 22	68 ± 30
	Hypoxia	48 ± 17	83 ± 30	76 ± 30
	Hypoxia + Carbo	41 ± 20	63 ± 31	43 ± 19
**LDH**	Rest	89.79 ± 15.55	131.39 ± 39.45	111.73 ± 18.55
	Exercise	91.39 ± 16.65	102.82 ± 17.08	134.03 ± 14.25
	Recovery	113.94 ± 39.22	148.01 ± 32.25	107.46 ± 37.16

Total CK (μmol/mL), CK-MB (μmol/mL) and LDH (μmol/mL) from plasma at rest, after exercise and after recovery, in normoxia, hypoxia and hypoxia + carbohydrate for *n* = 7 volunteers. Values shown represent the mean ± SD. Two-way ANOVA and Tukey’s post hoc tests, *p* < 0.05. ^a^ Different in relation to at rest.

**Table 3:** Serum concentration of IL-6, TNF-α and IL-10 and TNF-α/IL-10 at rest, after exercise and after recovery in normoxia, hypoxia and hypoxia + carbo.

	Condition	Rest	Exercise	Recovery
**IL-6**	Normoxia	1.13 ± 0.5	3.15 ± 1.64 ^a^	3.52 ± 0.5 ^a^
	Hypoxia	1.17 ± 0.14	3.30 ± 0.85 ^a^	2.87 ± 0.99 ^a^
	Hypoxia + Carbo	0.66 ± 0.32	2.21 ± 0.59 ^a^	1.83 ± 0.46
**TNF-α**	Normoxia	1.86 ± 0.30	4.49 ± 1.38	2.92 ± 1.57
	Hypoxia	1.82 ± 0.79	3.93 ± 2.55	3.65 ± 1.24
	Hypoxia + Carbo	2.65 ± 1.25	6.22 ± 1.89 ^a^	5.36 ± 5.59 ^a^
**IL-10**	Normoxia	5.74 ± 3.52	6.78 ± 1.46	9.19 ± 5.41
	Hypoxia	4.83 ± 2.18	8.02 ± 1.38	8.63 ± 1.66
	Hypoxia + Carbo	4.49 ± 1.58	6.38 ± 2.84	5.71 ± 2.70
**TNF-α/IL-10**	Normoxia	0.50 ± 0.39	0.68 ± 0.25	0.36 ± 0.21
	Hypoxia	0.42 ± 0.19	0.47 ± 0.23	0.44 ± 0.19
	Hypoxia + Carbo	0.63 ± 0.30	1.31 ± 1.08	0.84 ± 0.69

IL-6 (pg/mL), IL-10 (pg/mL) and TNF-α (pg/mL) from plasma at rest, after exercise and recovery, in normoxia, hypoxia and hypoxia + carbohydrate for *n* = 7 volunteers. Values shown represent the mean ± SD. Two-way ANOVA and Tukey’s post hoc tests, *p* < 0.05. ^a^ Different in relation to at rest.

**Table 4:** Serum concentrations of IL-4, IL-2/IL-4 and IL-2 at rest, after exercise and after recovery in normoxia, hypoxia and hypoxia + carbo.

	Condition	Rest	Exercise	Recovery
**IL-2**	Normoxia	2.80 ± 0.93	4.62 ± 1.62	4.40 ± 3.13
	Hypoxia	1.74 ± 1.18	3.14 ± 1.30	0.73 ± 0.54
	Hypoxia + Carbo	0.14 ± 0.20	0.39 ± 0.54	3.60 ± 4.65 ^a,b^
**IL-4**	Normoxia	1.39 ± 1.08	0.63 ± 0.48	0.42 ± 0.24
	Hypoxia	0.84 ± 0.94	0.41 ± 0.24	1.39 ± 2.42
	Hypoxia + Carbo	0.63 ± 0.30	0.38 ± 0.20	0.52 ± 0.48
**IL-2/IL-4**	Normoxia	3.50 ± 0.3	11.25 ± 7.82	13.11 ± 11.54
	Hypoxia	3.49 ± 0.24	9.20 ± 5.37	2.06 ± 2.05
	Hypoxia + Carbo	0.21 ± 0.22 ^c,d^	1.25 ± 1.75	11.84 ± 15.38 ^a,b^

IL-2(pg/mL), IL-4(pg/mL) and IL-2/IL-4 from plasma at rest, after exercise and recovery, in normoxia, hypoxia and hypoxia+ carbohydrate for *n* = 7 volunteers. Values shown represent the mean ± SD. Two-way ANOVA and Tukey’s post hoc tests, *p* < 0.05. ^a^ Different in relation to at rest; ^b^ different in relation to exercise, ^c^ different in relation to normoxia and ^d^ different in relation to hypoxia.

**Table 5:** Serum concentration of glutamine and glucose at rest, after exercise and after recovery in normoxia, hypoxia and hypoxia + carbo.

	Condition	Rest	Exercise	Recovery
**Glutamine**	Normoxia	0.56 ± 0.38	0.26 ± 0.02	0.24 ± 0.03 ^a^
	Hypoxia	0.26 ± 0.03 ^a^	0.31 ± 0.04	0.23 ± 0.02
	Hypoxia + Carbo	0.29 ± 0.08	0.39 ± 0.35	0.24 ± 0.03
**Glucose**	Normoxia	4.68 ± 0.41	4.61 ± 0.33	4.76 ± 0.16
	Hypoxia	4.83 ± 0.36	5.07 ± 0.63	5.10 ± 0.25
	Hypoxia + Carbo	4.60 ± 0.63	5.00 ± 1.02	4.22 ± 0.66

Glutamine (nmol) and glucose (mg/dL) from plasma at rest, after exercise and recovery, in normoxia, hypoxia and hypoxia + carbohydrate for *n* = 7 volunteers. Values shown represent the mean ± SD. Two-way ANOVA and Tukey’s post hoc tests, *p* < 0.05. ^a^ Different in relation to at rest.
